# Risk of weight gain for specific antipsychotic drugs: a meta-analysis

**DOI:** 10.1038/s41537-018-0053-9

**Published:** 2018-06-27

**Authors:** Jacob Spertus, Marcela Horvitz-Lennon, Haley Abing, Sharon-Lise Normand

**Affiliations:** 1 Department of Health Care Policy, Harvard Medical School, 180 Longwood Avenue, Boston, MA 02115 USA; 2 Cambridge Health Alliance, Harvard Medical School, 1493 Cambridge Street, Cambridge, MA 02139 USA; 3 RAND Corporation 20 Park Plaza, Suite 920, Boston, MA 02116 USA; 4 Department of Biostatistics, Harvard T.H. Chan School of Public Health, 677 Huntington Avenue, Boston, MA 02115 USA

## Abstract

People with schizophrenia are at considerably higher risk of cardiometabolic morbidity than the general population. Second-generation antipsychotic drugs contribute to that risk partly through their weight gain effects, exacerbating an already high burden of disease. While standard ‘as-randomized’ analyses of clinical trials provide valuable information, they ignore adherence patterns across treatment arms, confounding estimates of realized treatment exposure on outcome. We assess the effect of specific second-generation antipsychotics on weight gain, defined as at least a 7% increase in weight from randomization, using a Bayesian hierarchical model network meta-analysis with individual patient level data. Our data consisted of 14 randomized clinical trials contributing 5923 subjects (mean age = 39 [SD = 12]) assessing various combinations of olanzapine (*n* = 533), paliperidone (*n* = 3482), risperidone (*n* = 540), and placebo (*n* = 1368). The median time from randomization to dropout or trial completion was 6 weeks (range: 0–60 weeks). The unadjusted probability of weight gain in the placebo group was 4.8% across trials. For each 10 g chlorpromazine equivalent dose increase in olanzapine, the odds of weight gain increased by 5 (95% credible interval: 1.4, 5.3); the effect of risperidone (odds ratio = 1.6 [0.25, 9.1]) was estimated with considerable uncertainty but no different from paliperidone (odds ratio = 1.3 [1.2, 1.5]).

## Antipsychotics: Weight gain risk in schizophrenia

People taking a commonly prescribed antipsychotic to treat schizophrenia have an increased risk of weight gain. Sharon-Lise Normand of Harvard Medical School in the US and colleagues analysed data from 14 randomized control trials representing almost 6000 people taking one of three antipsychotic drugs for schizophrenia, or a placebo. Using a unique statistical approach, they found that people taking the drug olanzapine had the highest risk of weight gain. People taking paliperidone had only a small risk, while there was uncertainty regarding the drug riperidone. Antipsychotic drugs can often be taken for decades, so understanding their cummulative effects on patient health is crucial to improving quality of life and preventing premature mortality. The method used in this study is unique and could be used to analyse other aspects of drug use.

## Introduction

People with schizophrenia are at higher risk for obesity and cardiometabolic disorders including dyslipidemia, hypertension, type 2 diabetes, and cardiovascular disease (CVD) as a result of a number of factors, some inherent to the illness and associated lifestyle, and some related to the care they receive or fail to receive.^1–3^ This chronic morbidity adds to the disease burden of this vulnerable population, compounding their disability, worsening their quality of life, and causing premature mortality.^4,5^ A recent U.S. study found that patients with schizophrenia have a three- to four-fold increased risk of dying compared with the general population, with CVD contributing the most to this excess mortality.^6^ Antipsychotic drugs, the mainstay of treatment for people with schizophrenia, contribute to this risk and represent an important target for prevention efforts.^7–12^ It is therefore critical to understand antipsychotic drug risks, particularly those associated with some frequently used second-generation antipsychotics (SGAs). Several developments, including a U.S. Food and Drug Administration class warning on the metabolic risks of SGAs and the introduction of metabolically safer drugs, have had some impact on prescribing patterns in the U.S.^13,14^ However, SGAs with established or uncertain metabolic effects remain popular, even among patients with excessive weight and cardiometabolic disorders.

The evidence on antipsychotics’ cardiometabolic effects has been developed over the course of several decades.^8,15–26^ Some of the evidence comes from secondary data analyses of U.S. and U.K. administrative and medical databases, while others come from randomized controlled trial data. The studies that have contributed to this evidence are quite heterogeneous in their sample sizes and length of follow-up periods; case-definition (from schizophrenia-only to anyone prescribed antipsychotics); episode-construction methods; and for the naturalistic designs, definition of the treatment groups.^16,27^ Despite variable methodology and quality, there is substantial evidence suggesting that several frequently used SGAs are associated with significant metabolic risk, consistently highest for olanzapine and clozapine.^9,28–30^

A larger proportion of the evidence on SGA-related weight changes comes from randomized trials. A meta-analysis of randomized trials conducted between 1955 and 2012 that evaluated efficacy and tolerability of 15 antipsychotics, both first generation antipsychotics (FGA) and SGAs, found that olanzapine, followed by zotepine and clozapine were associated the highest risk for weight gain, followed in descending order by quetiapine, risperidone, and paliperidone.^31^ Only three drugs, haloperidol, one of the most frequently used FGAs, and two SGAs, ziprasidone and lurasidone, had risk of weight gain no different from placebo. These results are in keeping with other meta-analyses of randomized trials.^30,32^

Despite the substantial health burden associated with SGAs, several features of the metabolic risk of SGAs remain poorly understood. Little is known about the impact of intensity of exposure, that is, the cumulative dose over the length of treatment, on the likelihood of adverse metabolic outcomes.^33^ The standard of care for patients with schizophrenia includes receipt of maintenance treatment with antipsychotic drugs, which for most patients, means antipsychotic treatment for decades if not for life.^34^ Knowledge of the risks associated with intensity of exposure may affect recommendations and practice, particularly because the severity of the type of symptoms most effectively controlled by antipsychotics tends to abate over time.^35^ Gaining knowledge of the dose dependency of risk is also important from a public health perspective given the common practice of using doses on the higher end of the recommended range or above range for patients who are not responding to treatment.^36^ Finally, some of the dramatic growth in SGA utilization in the last decades has been driven by their frequent off-label use to manage non-psychotic conditions, presumably under the assumption that short exposures or small doses of antipsychotics may be safer than prolonged exposures at schizophrenia-tailored recommended doses. A better understanding of the risks of these drugs might impact off-label use given that the risk/benefit profile is by necessity worse for conditions lacking sufficient evidence of benefit.

To fill these gaps, we capitalize on a unique dataset and a method that analyzes observed cumulative exposure, accounting for dose, dropout, and adherence on a continuous exposure scale. We use a Bayesian hierarchical framework to analyze participant-level data obtained from the Yale Open Data Access (YODA) Project consisting of 13 RCTs conducted by Janssen Pharmaceuticals along with data from the Clinical Antipsychotic Trials of Intervention Effectiveness (CATIE) sponsored by the National Institute of Mental Health (NIMH).^37–49^ Using cumulative dose, we compare the risk of weight gain of three SGAs, paliperidone, olanzapine, and risperidone, evaluated on nearly 6000 randomized subjects.

## Results

We identified 5923 patients, 23% randomized to placebo, 59% randomized to paliperidone, 9% to risperidone, and 9% to olanzapine (Table 1) in studies conducted between 2001 and 2008. The number of subjects analyzed from each study varied from 114 to 749. Information of cumulative dose actually taken (including adherence) was available in 9 of the 14 trials; in 6 trials antipsychotics were delivered by injections. The median (maximum) total cumulative exposures were 2 (21.3), 0.6 (4.8), and 0.8 (5.6) 10 g CPZ equivalent units across trials for paliperidone, risperidone, and olanzapine, respectively. In trials with pill count information daily adherence to oral medication was modest, with a median of 78%, 25th percentile of 67%, and 75th percentile of 100%. In terms of participants, 37% were female, 64% white, with a mean (standard deviation) age of 40 (12) years. While key confounders were typically balanced well by randomization within studies, they often varied across studies. For example, subjects in study 2 were considerably older on average than in any of the other studies. All treatment groups were compared pairwise with all other treatment groups within at least one study with the exception of risperidone and placebo—risperidone was administered only in active controlled trials.

**Table 1:** Trial characteristics, exposure, baseline participant covariates, and outcomes across trials and treatment groups

Study	Duration	Total subjects	Antipsychotic	Total exposure: median (max)	% with weight gain drugs	Age: mean (SD)	PANSS: mean (SD)	% White	% Black	% Female	Outcome: % with 7% weight gain
					Baseline characteristics						
1	Variable (≤11 mts)	206	Paliperidone: 50.5%	3.06 (21.30)	9.6	39 (11)	92.9 (11.2)	60	8	44	18.27
			Placebo: 49.5%	0	10.8	37 (10)	94 (11.1)	60	9	37	11.76
2	6 weeks	114	Paliperidone: 66.7%	2.09 (4.50)	6.6	70 (5)	91.8 (9.7)	99	0	74	0
			Placebo: 33.3%	0	2.6	69 (3)	94.3 (9)	100	0	71	2.63
3	6 weeks	627	Olanzapine: 20.1%	0.84 (1.04)	2.4	36 (11)	93 (10.7)	87	0	54	22.22
			Paliperidone: 59.8%	2.46 (4.08)	2.1	37 (11)	94 (11.1)	86	0	46	10.93
			Placebo: 20.1%	0	2.4	37 (11)	94.1 (10.7)	84	0	48	3.17
4	6 weeks	438	Olanzapine: 24.9%	0.56 (1.92)	13.8	40 (11)	94.9 (12.3)	42	0	19	17.43
			Paliperidone: 51.1%	1.68 (3.84)	13.8	42 (10)	93.2 (11.7)	41	0	31	10.27
			Placebo: 24%	0	21.9	42 (11)	93.5 (11.9)	48	0	22	5.71
5	6 weeks	614	Olanzapine: 20.7%	0.84 (0.94)	4.7	37 (10)	93.4 (12.2)	48	23	24	19.69
			Paliperidone: 59.3%	2 (4.58)	11.5	37 (11)	92.6 (12.6)	49	21	36	10.99
			Placebo: 20%	0	8.9	37 (11)	93.8 (12.6)	51	21	30	5.69
6^a^	6 weeks	316	Pali: 66.1%	3.52 (8.00)	19.6	37 (10)	94.2 (12.9)	43	22	34	6.70
			Placebo: 33.9%	0	19.6	37 (11)	91.6 (12.5)	50	19	37	1.87
7^a^	6 weeks	310	Paliperidone: 69.4%	3.28 (4.00)	24.2	38 (9)	92.2 (13.5)	52	18	45	6.98
			Placebo: 30.6%	0	28.4	38 (10)	91.7 (12)	52	18	41	1.05
8^a^	18 months	342	Olanzapine: 50%	0.84 (3.9)	13.5	41 (11)	74.3 (19)	65	32	27	11.11
			Risperidone: 50%	0.42 (1.23)	20.5	41 (11)	76.8 (16.8)	66	30	27	5.26
9^b^	Recurrence prevention	408	Paliperidone: 50.2%	1.78 (6.67)	10.7	39 (11)	70 (18.3)	65	18	47	5.85
			Placebo: 49.8%	0	10.8	39 (11)	70.6 (17.6)	66	18	45	2.46
10^b^	53 weeks	748	Paliperidone: 50.7%	4.33 (8.50)	9	41 (12)	82 (12.7)	92	0	43	13.19
			Risperidone: 49.3%	0.38 (4.78)	8.4	41 (12)	81.2 (13.4)	92	0	38	14.09
11^b^	13 weeks	387	Paliperidone: 75.5%	1.33 (4.00)	15.8	40 (11)	90.4 (11.3)	40	41	32	12.33
			Placebo: 24.5%	0	20	40 (11)	93.9 (12.9)	38	41	27	9.47
12^b^	13 weeks	515	Paliperidone: 75.5%	1.33 (2.67)	11.3	40 (11)	90.8 (12)	67	29	32	12.34
			Placebo: 24.5%	0	7.9	40 (11)	90.7 (12.3)	66	29	38	7.14
13^b^	13 weeks	651	Paliperidone: 74.8%	2 (4.00)	7.6	39 (11)	87.3 (11.5)	53	31	32	11.29
			Placebo: 25.2%	0	6.7	39 (11)	86.8 (10.2)	54	32	34	4.27
14^b^	9 weeks	247	Paliperidone: 66%	1 (2.00)	8	38 (10)	94.4 (11.5)	70	17	31	5.52
			Placebo: 34%	0	7.1	39 (11)	95.7 (12.7)	73	17	35	3.57
All studies		5923	Olanzapine: 9%	0.84 (3.9)	8.8	39 (11)	87.5 (17.1)	62	16	31	**17.07**
			Paliperidone: 58.8%	2 (21.3)	11.1	39 (12)	89.3 (13.8)	62	18	38	**10.40**
			Placebo: 23.1%	0	12.1	40 (12)	89 (15.1)	60	18	38	**4.82**
			Risperidone: 9.1%	0.40 (4.78)	12.2	41 (12)	79.8 (14.7)	84	9	34	**11.30**

Exposure is in 10 g chlorpromazine equivalent units (i.e., 100,100 mg chlorpromazine equivalents)

*WG* weight gain, *PANSS* positive and negative syndrome scale

^a^Trial measuring adherence through pill counts

^b^Trial measuring adherence with injectable treatment drugs

### As-randomized analysis

The as-randomized analysis yielded adjusted odds weight gain (relative to no drug) of 4.66 (95% credible interval (CrI): 2.62, 8.04) for olanzapine, 2.19 (95% CrI: 1.59, 2.97) for paliperidone, and 2.12 (95% CrI: 0.28, 9.65) for risperidone. These results assume the odds are the same regardless of total cumulative dose taken. Table 2 displays the additive average treatment effects for randomization to each drug compared to placebo. The probability of experiencing excessive weight gain increased by 12.6% (95% CrI: 6.2, 20) on average when a subject was on olanzapine, 6% (95% CrI: −2.3, 24.9) on risperidone, and 4.6% (95% CrI: 2.6, 6.7) on paliperidone. In terms of ranking, olanzapine had an 88% chance of having the largest effect on the probability of excessive weight gain, risperidone had an 11% chance and paliperidone had less than a 1% chance.

**Table 2:** Excess risk (%) of at least 7% weight gain associated with changes in doses and antipsychotics

Antipsychotic	Paliperidone	Risperidone	Olanzapine
Dose, mg	0 to 300	0 to 80	0 to 420
Excess risk, %	3.2 (1.5, 5.3)	1.3 (−2.3, 6.1)	12.2 (1.4, 27.2)
%^a^	4.6 (2.6, 6.7)	6 (−2.3, 24.9)	12.6 (6.2, 20)
Dose, mg	300 to 975	80 to 427	420 to 707
Excess risk, %	14.3 (5.5, 26.9)	13 (−3, 70.3)	16.7 (1.2, 34.9)
%^a^	0	0	0
Dose, grams CPZ		0 to 10	
Excess risk, %	1.4 (0.7, 2.3)	4.5 (−3.8, 24.4)	16.1 (1.7, 36.4)
%^a^	4.6 (2.6, 6.7)	6 (−2.3, 24.9)	12.6 (6.2, 20)
Dose, grams CPZ		0 to 20	
Excess risk, %	3.2 (1.5, 5.3)	12.6 (−5, 70)	46.8 (3.9, 83.6)
%^a^	4.6 (2.6, 6.7)	6 (−2.3, 24.9)	12.6 (6.2, 20)

The first two rows provide casual estimates across doses but within treatment drugs, in terms of their original scale (milligrams of each drug). Thus, the excess risk associated with an increase in cumulative dose from 0 mg to the 50th percentile and from the 50th to 90th percentile of each drug is shown. Estimates can be compared across drugs when reported in CPZ equivalent units as in the 3rd and 4th row

^a^Denotes the as-randomized estimates that assume no dose effect

### As-treated intensity of exposure analysis

In terms of estimated parameters, the odds of weight gain for an increase of 10 g CPZ equivalent unit were 4.99 (95% CrI: 1.36, 15.33) for olanzapine, 1.31 (95% CrI: 1.16, 1.50) for paliperidone, and 1.62 (95% CrI: 0.25, 9.14) for risperidone. The within-trial estimates are available in our Supplementary appendix. Although the interval estimate for the odds ratio for risperidone includes one, there was an 81% chance that risperidone increases the probability of ≥7% weight gain. Furthermore, we find an 89% chance that olanzapine has the largest effect on the risk of ≥7% weight gain, risperidone a 10% chance, and paliperidone a 1% chance.

Table 2 shows average treatment effects at various doses representing either observed percentiles of exposure within different drugs or fixed CPZ equivalent doses. The additional risk of increasing dose from 0 to the 50th percentile was relatively low for paliperidone and risperidone compared to moving from the 50th to 90th percentiles, likely because the dose increased considerably for these drugs between their upper exposure percentiles: from 300 to 975 mg for paliperidone and from 80 to 427 mg for risperidone. We cannot directly compare average treatment effects across drugs in terms of their original doses, but we can in terms of fixed CPZ equivalent doses. At both 10 g CPZ and 20 g CPZ, olanzapine delivers by far the largest increase to the risk of weight gain at 16.1% and 46.8%, respectively. Again there is considerable uncertainty around risperidone, and paliperidone delivers a small increase in the probability of weight gain.

**Fig. 1:**
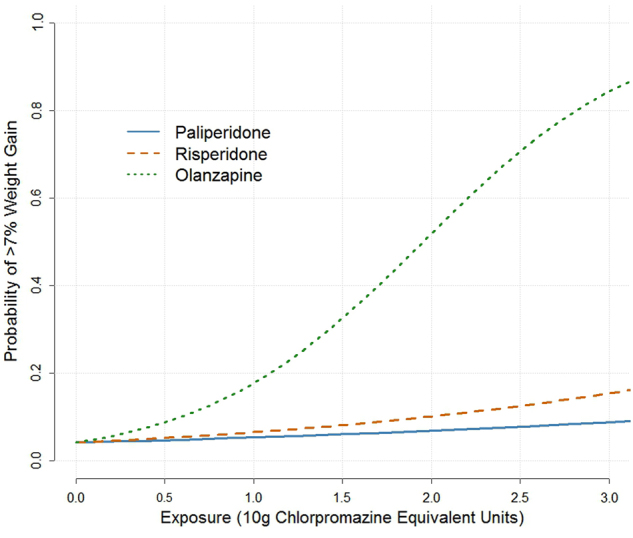
Average dose–response curves. Olanzapine (green/dotted), risperidone (orange/dashed), and paliperidone (blue/solid). *X*-axis is exposure in 10 g chlorpromazine equivalent units. *Y*-axis gives probability of excessive weight gain (≥7%). The intercept is based on average treatment-free response across the sample. For clarity, we do not plot the credible intervals around the curves

Dose–response curves based on average intercepts and posterior mean treatment slopes are plotted in Fig. 1.

### Sensitivity analysis

Our results were robust to a number of different analysis decisions, including the common scale for exposure (chlorpromazine equivalents or PORT recommended ranges) and maximum duration of exposure (in weeks) for subjects included in the analysis. We also tested a more complex model which included adherence as a study-level predictor of the treatment slopes. We found that the interactive effect of an adherence indicator with the slope had a small, insignificant coefficient. Furthermore, a comparison using the leave-one-out information criterion favored the simpler model over one that included adherence, so we did not account for it in our final estimates.

## Discussion

We sought to combine information across randomized trials to assess the effect of actual exposure to three frequently used antipsychotic drugs on the likelihood of excessive weight gain, which as others we defined as weight gain of 7% or more from baseline.^32^ Although our findings are consistent with other evidence that olanzapine is associated with a higher risk of excessive weight gain relative to other antipsychotics,^31,37,40,43,50^ we add to the limited comparative evidence on risperidone versus paliperidone^31,48^ and expand the evidence base on the metabolic effects of the intensity of exposure of antipsychotics by providing the first RCT-based analyses of these associations. These findings are an important contribution to the evidence base on the safety of antipsychotics. These drugs are the mainstay of the treatment of schizophrenia as well as other serious mental illnesses, and they are used for prolonged periods of time, often at doses that exceed recommended doses.^34,36^ Thus, it is critical to understand the effect of cumulative dose on the drugs’ metabolic effects. While we did not directly assess the drugs’ impact on coronary artery disease, excess weight is associated with metabolic syndrome, dyslipidemia, hypertension, Type 2 diabetes, and artherosclerosis, all risk factors for coronary artery disease. Nevertheless, we caution the reader that these results do not speak directly to the drugs’ effects on coronary artery disease or other cardiovascular disorders.

The Bayesian hierarchical approach permitted an accounting of the heterogeneity between trials while generating posterior summaries that have great relevance to decision makers. For example, we were able to quantify the probability that each treatment was the most likely to cause the adverse outcome. The benefits of a Bayesian framework for network meta-analysis have been widely discussed elsewhere.^31,51^

Furthermore, by analyzing a continuous treatment derived from observed exposure incorporating dropout, dosing, and (in some trials) adherence, we estimated a more meaningful and externally valid causal effect than a traditional comparison of mean outcomes between as-randomized treatment groups, which provides an unbiased effect estimate of treatment assignment.^52^ To contrast our new approach to typical analyses, we conducted an as-randomized meta-analysis using treatment assignment indicators. Although the interpretation of parameter estimates from these two approaches is different, we found that the estimated parameters aligned in terms of ranking with olanzapine emerging as the clear worst while risperidone and paliperidone had similar effect sizes. Our results also aligned with findings from past as-randomized studies, which should strengthen the conclusions drawn from both methods.^31^ However, relating outcome to a continuous exposure is more relevant to clinical practice. For example an odds ratio of 4.2 for randomization to olanzapine versus placebo may not be that meaningful in a non-randomized setting, while an odds ratio of 5 for a 10 g chlorpromazine equivalent dose (or 500 mg) of olanzapine has direct significance. Supplementing an as-randomized analysis with a dose–response model can remove bias caused by differential adherence or dropout, and yield more relevant information for practitioners even if such bias does not exist.

We noted that the method we proposed is quite general and could be applied to any dose–response question where it is beneficial to treat exposure as a continuous variable. It is simple to implement such an analysis within individual RCTs by ignoring the hierarchical piece of the proposed model. In general, any drug, primary outcome, or side-effect can be analyzed in this manner. Even continuous, count, or categorical dependent variables can be readily incorporated by simple alterations to our method within the generalized linear model framework.

Because actual exposure was not randomized, our analysis was fundamentally an observational study even though it used RCT data. While the estimates obtained are more externally valid and relevant to decision makers, confounding can bias a simple regression of outcome on treatment. Thus, we adjusted for potential confounders, selected based on subject matter expertise, and assumed that there were no additional unmeasured confounders that may have biased our results. The additional modeling and assumptions required represent a tradeoff that should hinge on careful consideration of the problem at hand, including potential threats to the external validity of RCT results, the availability of measured confounders, and the decisions that will eventually be made using the insights from the study.

Another challenge with comparing treatment drugs under continuous exposure is putting them on the same scale. Because potencies vary widely between drugs and our ultimate goal is to compare drugs to each other, it is not reasonable to use the original doses as a measure of continuous exposure. We chose to use well-established chlorpromazine equivalents, which standardizes doses based on the antipyschotic drugs efficacy, as our main exposure measure and converted original doses accordingly. However this quantity is itself estimated and may not be the best conversion scale to use. As a sensitivity analysis we checked our results using the dose ranges recommended by the PORT guidelines.^34^ We found our results to be robust under these different exposure conversions, but future researchers should be aware of this challenge and conduct similar checks.

Because we did not have adherence information in five trials with orally administered drugs, we had to assume perfect adherence in these trials. This could potentially overestimate the risk of weight gain, but adherence was generally adequate in our trials where it was measured and variation in exposure due to different prescribed doses and dropout were taken into account in all trials.

Our methods could be improved by allowing for non-linear treatment effects. It is likely that the log-odds effect of these drugs are not constant, but higher during the lower initial doses and become less steep or plateau at higher doses. Modern techniques could more finely capture these distinctions. Therefore in addition to addressing the problem of scaling continuous drug exposures, future work should continue to develop flexible and intuitive non-parametric treatment effect estimators.

## Methods

### Data and population

Our data consisted of participant-level data from 13 randomized clinical trials of antipsychotic drugs obtained from the Yale Open Data Access Project (YODA). Janssen Pharmaceuticals conducted 13 of these trials, and while YODA provides data from 17 Janssen trials, we excluded 3 trials because they involved patients with bipolar disorder. We refer to the 13 trials collectively as the *YODA* data. The length of these trials varied: six 6-week, one 9-week, three 13-week, one 53-week, and two variable length trials. The final trial was the Clinical Antipsychotic Trials of Intervention Effectiveness (CATIE), an 18-month trial obtained from the National Institute of Mental Health. The CATIE trial was 18 months in duration and drug doses were relatively high compared to the 13 YODA trials. We included CATIE subjects who agreed to provide genetic data as this dataset is of considerably higher quality, and participants did not differ systematically from those who did not provide genetic data. We analyzed the data for participants who received a dose of study medication and provided post-baseline safety data (e.g., vital measurements).

All participants were adults aged between 18 and 84, diagnosed with schizophrenia.

### Primary outcome

We subtracted each participant’s weight at study termination (or at last available observation) from their baseline weight measured at treatment initiation. We then identified individuals who experienced a weight gain of 7% or more. The 7% threshold is typically used in randomized trials to assess the incidence of excessive weight gain.^32^ All but four studies reported actual weights; the four studies grouped weight in categories. For these four studies, we used the median value (e.g., ‘60–65 kg’ became 62.5 kg), and used these derived numerical weights when computing the binary outcome.

### Treatments and exposures

We focused on three SGAs, paliperidone, olanzapine, and risperidone, and make use of placebo arms. We confined our analysis to treatment drugs that were measured in two or more of our trials, restricting the CATIE trial participants to those who received either risperidone or olanzapine. We operationalized intensity of exposure through a measure of cumulative dose taken, defined as the total dose taken in 100 mg of chlorpromazine (CPZ) equivalent units over the course of the double-blind phase in each trial.^53^ This implied that each participant contributed one (point) exposure measurement that we associate with the primary outcome. Calculation of exposure varied by study, with some trials providing relatively accurate measures based on pill counts or injections, and others providing prescribed daily dose over fixed periods along with time to dropout. In the latter case, we derived cumulative dose by multiplying prescribed dose by the number of days in each period. Because these studies did not report adherence through actual pill counts or injections taken, the resulting cumulative dose measure provided an upper bound on true exposure. Thus our exposure measure accounted for prescribed dose, trial duration, and dropout in all trials, and adherence as well when it was available.

We combined treatment groups that represented different doses of the same molecule. For example, although the original study groups in study 4 included two paliperidone groups defined by different daily doses (6 mg and 12 mg), we treated both as paliperidone with the dosing difference ultimately reflected in our continuous measure of total exposure. We also combined evidence from oral and injectable versions of the same drugs. Specifically, exposure to both oral paliperidone extended-release tablets and paliperidone palmitate injections were measured as exposure to paliperidone, and exposure to risperidone tablets or Risperdal consta injections were measured as exposure to risperidone. Finally, we rescaled exposure units such that each unit of exposure represents 100,100 mg (or 10 g) CPZ equivalent doses of a given treatment drug.

In our primary analyses, we focused on exposures up to 13 weeks. The CATIE study was much longer than the other trials and is likely to contribute high leverage points. Because weight and exposure were measured at every time point in the CATIE data, we truncated the CATIE data to participant’s 2nd visit during the double-blind phase, approximately around 8-weeks from randomization. We included all subjects participating in study 1 and study 10, which were the only YODA trials greater than 13 weeks. We could not truncate these trials because weight data were primarily collected at trial initiation and termination.

### Other confounders

Additional baseline participant-level covariates, including age, sex, race, body mass index (BMI), use of drugs causing weight gain, and positive and negative syndrome scale (PANSS, range: 30–210 with higher values indicating more severity), were used to adjust for between-arm differences. In each trial, baseline measurements were those taken at the beginning of the double-blind phase. Indicators for general heart abnormalities and use of drugs causing weight loss were also available at the patient level, but we did not include them because prevalence was very low (2.6% and 0.9%, respectively) and a preliminary analysis of bivariate association with outcome revealed no relationship. We also recorded a study-level indicator for whether the study recorded actual pills taken, either by nature of dispensing long-term injectable medication or by gathering pill counts for daily oral medications. There was a small amount of missing data in some of the trials and a single complete dataset was imputed using predictive mean matching.

### Statistical model

We utilized a Bayesian hierarchical generalized linear model to combine the data from the 14 clinical trials.^51,54,55^ This framework is especially useful for network meta-analysis, which provides a quantitative method for integrating participant-level data from all available comparative study arms.^51,54,56,57^ We borrowed information from randomized treatment arms from different studies and different exposure durations to better estimate the exposure–outcome relationship for each drug as well as for no drug. We made a number of assumptions needed to interpret the dose–response curves causally. First, we assumed that the particular drug (and dose) taken by one participant in a trial did not cause weight gain in a subject who participated in a different trial, and that participants who received a fixed dose of the same drug received the same treatment. This assumptions seems reasonable. Second, we assumed that all patients could have received any SGA at any observed dose. While randomization ensured the first part of this assumption within and across trials, the any observed dose is not guaranteed. Some trials dosed more intensely in terms of CPZ equivalents than others. For example, the maximum exposure to paliperidone was higher than any of the other SGAs. We thus extrapolated the exposure–outcome relationships for all SGAs other than paliperidone at high exposures. It may be plausible that for a specific participant, had they been assigned to a different drug, the actual cumulative dose taken could be different from that taken on the drug they did take. We conducted a sensitivity analysis to determine how robust our findings were to this assumption. We also assumed the biological effect of those assigned to placebo and those assigned to active drugs but did not take any were similar. Finally, we assumed no unmeasured confounding such that, after adjusting for baseline confounders, our estimate of the causal effect is unbiased. This assumption is untestable and must be assessed within the specific design based on confounder availability.

We estimated two distinct models: an as-randomized analysis with indicators for treatment group assignment and an as-treated regression of the odds of weight gain associated with a 10 g CPZ increase by making use of continuous cumulative dose. Both models included baseline potential confounding variables. The as-randomized analysis modeled the log-odds of weight gain as a function of treatment assignment, confounders, and study-specific random effects for each treatment arm. These study-specific effects were drawn from a common multivariate normal distribution across trials. This model produced odds ratios characterizing the odds of weight gain when randomized to a specific SGA (regardless of dose) relative to the odds when randomized to placebo; the as-treated regression provided the odds of weight gain for each 10 g CPZ increase in dose. For both models, the treatment-free response was also modeled hierarchically. In the as-randomized analysis, active controlled trials essentially borrowed information from placebo-controlled studies in order to estimate their specific intercept because these trials had no placebo arm. This gave estimates of the treatment effects both within and across trials, the latter being of more interest in this context because it reflects the evidence provided by pooling available sources of treatment effect information. Further details on the models we fit are available in our Supplementary appendix.

For the as-randomized analysis, we reported adjusted odds ratios and 95% credible intervals (CrI) of the relationship of random assignment to a particular SGA relative to placebo as well as the probability that each drug has the largest effect on the probability of excessive weight gain. For continuous as-treated cumulative dose, we reported the odds of an increase of 10 g CPZ units relative to no increase for each specific SGA as well as the odds of weight gain associated with differences in particular cumulative doses.

In addition to coefficients, we calculated average treatment effects on the probability scale in terms of the additional percent chance of experiencing ≥7% weight gain. For the as-randomized analysis this gave the additional risk compared to placebo if randomized to one of the three treatment drugs. For continuous as-treated exposure many potential average treatment effects can be calculated. We considered the effect of increasing a drug from 0 dose to the 50th percentile of observed dose and also from the 50th to 90th percentile of observed dose. We also assessed the effect of moving from 0 dose to a fixed 10 g or 20 g CPZ equivalent dose. The former is primarily interesting for comparing risk at different doses of the same drug and highlights the dose dependency of weight gain risk, but should not be used for comparisons across drugs. The fixed CPZ equivalent average treatment effects better facilitate comparison among drugs because CPZ equivalents are on the same scale by normalizing their potencies.

To estimate these average treatment effects, we calculated the predicted response for each patient at an appropriate baseline (e.g., each randomized to placebo or each receiving no dose of any drug) and also for a fixed given treatment (e.g., each randomized to olanzapine or each given a 10 g CPZ equivalent dose of olanzapine). The difference between the average response on active treatment (or increased dose) and the average response at baseline was the average treatment effect.

We also plot dose–response curves by calculating an average treatment-free response across all patients, and then drawing a curve continuously across doses up to a 30 g CPZ equivalent using the posterior mean log-odds treatment effect slope.

For all quantities, the full posterior distribution with corresponding point estimates and credible intervals was obtained using Bayesian computation, elaborated further in our Supplementary appendix.

### Sensitivity analysis

We examined the robustness of our results on different exposure scales derived from the Schizophrenia Patient Outcomes Research Team treatment recommendations.^34^ That review recommended daily dose ranges of 3–15 mg for paliperidone, 10–20 mg olanzapine, and 2–8 mg risperidone. We conducted two additional analyses, one with exposure represented in units of daily doses at the low end of the range (3 mg paliperidone, 10 mg olanzapine, and 2 mg risperidone) and one with exposure as daily doses at the high end (15 mg paliperidone, 20 mg olanzapine, and 8 mg risperidone). Because trials varied in length, we also assessed the sensitivity of our results if we restricted the patient populations to a reasonable upper range. We did this by dropping participants who stayed in longer than 12 weeks. Finally, because trials varied by how exposure was collected, we determined whether mode of exposure collection (pill counts or injections versus not) modified the relationship with the outcome. We estimated a model where we interacted the treatment exposure with exposure collection mode, and compared models with and without the interaction term using the leave-one-out information criterion.^55^

### Code availability

Details on how we fit the model are available in our Supplementary appendix, along with the R script used to fit the final model.

### Data availability

This study, carried out under YODA Project No. 2015–0678, used data obtained from the Yale University Open Data Access Project, which has an agreement with Janssen Research & Development, L.L.C. The interpretation and reporting of research using this data are solely the responsibility of the authors and does not necessarily represent the official views of the Yale University Open Data Access Project or Janssen Research & Development, L.L.C. The CATIE data used in this paper reside in the NIH-supported NIMH Data Repositories NIMH Data Repositories DOI: 10.15154/1373363]; Principal Investigators of original data: J. Lieberman (N01-MH090001) and P. Sullivan (R01-MH074027).

The CATIE data is available from the National Institute of Medical Health Repository and Genomics Resource (https://bioq.nimhgenetics.org/studies/?studyId=20). The Janssen Trials are available from the Yale Open Data Access Project (http://yoda.yale.edu/multiple-ncts-optics-trial-bundle).

### Disclaimer

The content is solely the responsibility of the authors and does not necessarily represent the official views of Harvard Catalyst, Harvard University and its affiliated academic healthcare centers, or the National Institutes of Health.

## Electronic supplementary material

R Code

Supplementary Appendix: Risk of weight gain for specific antipsychotic drugs: A meta-analysis
