# Supplementary material for: Risk of weight gain for specific antipsychotic drugs: a meta-analysis
Source: NPJ Schizophr. 2018 Jun 27;4:12. doi: 10.1038/s41537-018-0053-9 (PMC6021430; doi:10.1038/s41537-018-0053-9)
Supplement: Supplementary file 2 — Supplementary Appendix: Risk of weight gain for specific antipsychotic drugs: A meta-analysis [file 41537_2018_53_MOESM2_ESM.pdf]

Supplementary Appendix: Risk of weight gain for specific antipsychotic drugs: A meta-analysis

Jacob Spertus, Marcela Horvitz-Lennon, Haley Abing, and Sharon-Lise Normand

1 Estimation Details

As-Randomized Analysis We set Y_{ij} to 1 if the ith of n_j participants in trial j of J trials gained at least 7% of their baseline weight and 0 otherwise. Each participant has a $K \times 1$ vector of indicators $\boldsymbol{Z}_{ij}$ where entry k is equal to 1 if the patient was randomized to drug k and 0 otherwise. For a patient assigned to placebo, all entries of $\boldsymbol{Z}_{ij}$ are equal to 0. Finally, each participant has a $p \times 1$ vector of baseline individual covariates $\boldsymbol{X}_{ij}$. We assume that Y_{ij} arises from a Bernoulli distribution specified as:

$$Y_{ij} \sim \text{Bernoulli}\left\{\text{logit}^{-1}(\alpha_{0j} + \boldsymbol{\psi}_j \boldsymbol{Z}_{ij} + \boldsymbol{\beta}\boldsymbol{X}_{ij})\right\}. \tag{1}$$

The $1 \times p$ parameter $\boldsymbol{\beta}$ represents covariate effects shared across trials; α_{0j}, the intercept, is the mean log-odds of at least a 7% weight gain for participants in trial j randomized to placebo; and the $1 \times K$ parameter vector $\boldsymbol{\psi}_j = [\psi_{j1}, ...\psi_{jK}]$ has kth element representing the the effect of *randomization* to antipsychotic k in trial j.

To make use of all the trial information, we assume that the trial-specific intercept α_{0j} and treatment effects $\boldsymbol{\psi}_j$ arise from a multivariate normal distribution with $1 \times (K+1)$ mean vector $[\mu_\alpha, \boldsymbol{\mu}_\psi] = [\mu_\alpha, \mu_{\psi_1}, ...\mu_{\psi_K}]$ (Equation (2)). The first term, μ_α, represents the overall treatment-free response across trials; the remaining K terms represent the change in log-odds of weight gain for each drug k across trials (μ_{ψ_k}). Between trial variability is accommodated through the $(K+1) \times (K+1)$ covariance matrix of trial-specific effects, denoted $\boldsymbol{\Sigma}$. We assume that covariance matrix can be decomposed into a diagonal matrix $\text{D}(\boldsymbol{\sigma})$ whose entries are defined by a length $(K+1)$ vector of standard deviations $\boldsymbol{\sigma}$ and a $(K+1) \times (K+1)$ correlation matrix $\boldsymbol{\Omega}$. The entries of $\boldsymbol{\sigma} = [\sigma_0, \sigma_1 ... \sigma_K]$ correspond to the standard deviations of the intercept and treatment effects across trials, and $\boldsymbol{\Omega}$ defines the correlation structure of those same parameters.[1]

$$\begin{bmatrix} \alpha_{0j} \\ \boldsymbol{\psi}_j^{\text{T}} \end{bmatrix} \overset{\text{iid}}{\sim} \mathcal{N}_{1+K}\left\{ \begin{bmatrix} \mu_\alpha \\ \boldsymbol{\mu}_\psi \end{bmatrix}, \boldsymbol{\Sigma} \right\} \text{ where } \boldsymbol{\Sigma} = \text{D}(\boldsymbol{\sigma})\boldsymbol{\Omega}\text{D}(\boldsymbol{\sigma}) \tag{2}$$

To include uncertainty about these relationships, several prior distributions are adopted for the parameters in Equations (1) and (2). We first rescale continuous confounders in $\mathbf{X}_{ij}$ to have standard deviation 1, assume that $\boldsymbol{\beta} \sim t_5(0, 2.5)$, and that the individual components of $\boldsymbol{\beta}$ are a-priori independent. The notation $t_5(0, 2.5)$ refers to a Student-t distribution with degrees of freedom 5, mean 0, and scale 2.5; it is weakly informative for logistic regression coefficients (see[2,4]). We adopt $\mu_\alpha \sim \mathcal{N}(0, 10)$, a relatively vague prior distribution for the average log-odds of at least 7% weight gain when taking no active drug across the trials and also assume that $\mu_{\psi_k} \sim t_5(0, 2.5)$. We assume half-$t_5(0, 2)$ distributions for the components of $\boldsymbol{\sigma}$ that are weakly informative but provide enough regularization to allow estimation even when there are small groups (for example, antipsychotics studied in only a few trials). For the correlation matrix, $\boldsymbol{\Omega} \sim \text{LKJ}(2)$, the Lewandowski, Kurowicka, Joe (LKJ)-correlation prior is used. This distribution is popular as a hierarchical correlation matrix in modern Bayesian computation packages.[1,3] Its single parameter controls how the correlation matrix $\boldsymbol{\Omega}$ concentrates *a priori* on the identity matrix, with a value of 1 indicating a flat prior on all correlation matrices, and a value larger than 1 favoring more concentration at the identity matrix and thus lower correlation values. Our setting of 2 can be considered weakly informative, which was important given some of our parameters (e.g. for risperidone) were estimated in few trials such that a completely vague prior may have led to an unstable posterior.

In our analysis, X_{ij} consists of standardized age, PANSS, and BMI, as well as indicators for female, race (Black, Asian, or Other), and whether the subject was on weight gain drugs at trial initiation so that $p = 8$. Finally, we have $K = 3$ different antipsychotics implying that Σ is a 4×4 covariance matrix.

As-treated Intensity of Exposure Analysis In addition to the data specified above, each participant has a $1 \times K$ observed cumulative dose taken vector $\boldsymbol{E_{ij}} = [E_{ij1}, ...E_{ijK}]$ where E_{ijk} represents the cumulative dose of antipsychotic k taken by trial termination. If a subject was not randomized to antipsychotic k, then necessarily $E_{ijk} = 0$. All entries of $\boldsymbol{E_{ij}}$ equal 0 if a subject was randomized to placebo. The outcome again arises from a Bernoulli with specification:

$$Y_{ij} \sim \text{Bernoulli}\left\{ \text{logit}^{-1}(\alpha_{0j} + \boldsymbol{\psi_j E_{ij}} + \boldsymbol{\beta X}_{ij}) \right\}. \tag{3}$$

The parameter ψ_{jk} represents a treatment and trial specific *slope* on the log-odds scale, while the elements of $\boldsymbol{\mu}_\psi$ quantify overall antipsychotic effect slopes. The interpretations of α_{0j} and the corresponding overall intercept μ_α are also differ from Equation (1) because it does not just capture the mean outcome in the placebo group but also the treatment-free response across treatment groups, i.e. what occurs on average when $E_{ijk} = 0$ for all k.

Exposures on the 10,000mg chlorpromazine equivalent scale had a wider range than the $\approx (-2, 2)$ range implied by normal standardization (see Table 1). As a result the $t_5(0, 2.5)$ priors assigned were especially vague for the treatment effect estimates, which is desirable. We used the brms R package to fit the model, drawing from the posterior distribution using Hamiltonian Monte Carlo simulation.[1] 3 chains were run, and after 400 burn-in iterations, 400 draws were kept from each for a total of 1200 samples used for inference. As diagnostics we visually inspected trace plots of the parameters and checked that all samples had a Gelman-Rubin 'r-hat' statistic lower than 1.1, which indicates that the chains had converged to the posterior. Effective sample sizes were also generally quite high and the chains had low autocorrelation, due to the efficiency of the Hamiltonian Monte Carlo algorithm.

2 Sensitivity Analysis Results

Overall treatment effect estimates were very similar when we truncated the participant population to subjects exposed for less than 12 weeks. The estimate (95% credible interval) for paliperidone was 1.4 (1.2, 1.6), risperidone was 1.8 (0.1, 24.7), and olanzapine was 5.7 (1.2, 17.7).

The model we fit allowing interaction with adherence produced no significant results, in the sense that neither the main effect of adherence nor the interaction terms of adherence with exposure had 95% credible intervals

that excluded 0. Specifically, on the log-odds scale the point estimate (95% Credible Interval) for interaction with paliperidone was 0.1 (-0.2,0.3), olanzapine was -1.3 (-2.8,0.8), and risperidone was 0.3 (-3.8, 4.2). Furthermore, a comparison to the standard fit using the leave-one-out information criterion indicated no significant improvement in overall fit when adding in adherence information.[4] Considering these statistics and in the interest of parsimony, we did not include adherence in our full analysis.

3 Within-Trial Hierarchical Results

The table below reports the posterior means and 95% credible intervals obtained from the as-treated intensity of exposure analyses. Here we present the complete within-trial results ($\exp \alpha_{0j}$ and $\exp \psi_{jk}$) as well as the overall results ($\exp \mu_\alpha$ and $\exp \boldsymbol{\mu}_\psi$; top row) estimated from our hierarchical continuous exposure model. For trials where a certain antipsychotic was not tested, the effect estimates is essentially just a draw from the appropriate posterior distribution.

	**Drug**			
**Study**	**None**	**Paliperidone**	**Risperidone**	**Olanzapine**
All	0.03 (0.02, 0.04)	1.31 (1.16, 1.5)	1.62 (0.25, 9.15)	4.99 (1.36, 15.33)†
Study 1	0.07 (0.04, 0.11)	1.05 (0.96, 1.13)	1.68 (0.05, 55.67)†	3.66 (0.11, 77.06)
Study 2	0.01 (0, 0.04)	1.15 (0.8, 1.56)	1.58 (0.05, 39.15)†	4.33 (0.15, 78.73)†
Study 3	0.03 (0.02, 0.04)	1.36 (1.15, 1.66)	1.64 (0.05, 44.33)†	8.13 (3.99, 18.71)
Study 4	0.04 (0.02, 0.06)	1.52 (1.22, 1.92)	1.55 (0.03, 52.54)†	9.35 (3.96, 25.37)
Study 5	0.03 (0.02, 0.04)	1.41 (1.19, 1.66)	1.6 (0.04, 47.81)†	6.76 (3.2, 14.17)
Study 6	0.02 (0.01, 0.03)	1.18 (1.01, 1.37)	1.51 (0.04, 40.09)†	4.56 (0.17, 79.11)†
Study 7	0.02 (0.01, 0.03)	1.38 (1.11, 1.75)	1.53 (0.04, 42.62)†	5.44 (0.39, 77.5)†
Study 8	0.04 (0.02, 0.07)	1.27 (0.88, 1.88)†	1.87 (0.47, 7.45)	2.2 (1.14, 4.32)
Study 9	0.02 (0.01, 0.03)	1.35 (1.12, 1.7)	1.55 (0.06, 26.84)†	5.15 (0.3, 53.45)†
Study 10	0.07 (0.05, 0.1)	1.17 (1.09, 1.26)	1.52 (1.16, 1.98)	4.32 (0.27, 71.74)†
Study 11	0.05 (0.03, 0.07)	1.34 (1.1, 1.69)	1.68 (0.07, 46.18)†	4.97 (0.31, 82.72)†
Study 12	0.04 (0.03, 0.07)	1.48 (1.19, 1.88)	1.64 (0.04, 87.66)†	5.97 (0.45, 101.77)†
Study 13	0.03 (0.02, 0.04)	1.44 (1.22, 1.72)	1.67 (0.07, 49.33)†	5.69 (0.33, 88.9)†
Study 14	0.02 (0.01, 0.04)	1.29 (0.95, 1.75)	1.74 (0.06, 88.14)†	5.19 (0.24, 82.39)†

Table A1: Posterior mean adjusted odds-ratio overall and within trials estimated from hierarchical continuous exposure model. †= drug not actually included in given trial.

4 References

1. Burkner, PC brms: Bayesian regression models using STAN. *Version 1.7.0* (2017).

2. Gelman, A Prior distributions for variance parameters in hierarchical models. *Bayesian Analysis*, **1**, 515–553 (2006).

3. STAN Development Team STAN modeling language users' guide and reference manual. *Version 2.14.0* (2016).

4. Gelman A, et al. *Bayesian Data Analysis, Third Edition*, (Chapman & Hall/CRC, New York, USA, 2014).
